# PTPMT1 protects cardiomyocytes from necroptosis induced by γ-ray irradiation through alleviating mitochondria injury

**DOI:** 10.1152/ajpcell.00466.2022

**Published:** 2023-05-08

**Authors:** Jing Yi, Liang Yue, Yuning Zhang, Ning Tao, Han Duan, Lin Lv, Yingxia Tan, Hua Wang

**Affiliations:** ^1^College of Life Science, Anhui Medical University, Hefei, People’s Republic of China; ^2^Department of Experimental Haematology, Beijing Institute of Radiation Medicine, Beijing, People’s Republic of China; ^3^Department of Stem Cell and Regenerative Medicine, Institute of Health Service and Transfusion Medicine, Beijing, People’s Republic of China; ^4^Department of Oncology, The Fifth Medical Center of Chinese PLA General Hospital, Beijing, People’s Republic of China; ^5^School of Life Sciences, Hebei University, Baoding, People’s Republic of China

**Keywords:** induced pluripotent stem cell-derived cardiomyocytes (iPSC-CMs), mitochondrial injury, necroptosis, protein tyrosine phosphatase, mitochondrial 1 (PTPMT1), radiation-induced heart disease

## Abstract

Radiation-induced heart disease (RIHD) progresses over time and may manifest decades after the initial radiation exposure, which is associated with significant morbidity and mortality. The clinical benefit of radiotherapy is always counterbalanced by an increased risk of cardiovascular events in survivors. There is an urgent need to explore the effect and the underlying mechanism of radiation-induced heart injury. Mitochondrial damage widely occurs in irradiation-induced injury, and mitochondrial dysfunction contributes to necroptosis development. Experiments were performed using induced pluripotent stem cell-derived cardiomyocytes (iPSC-CMs) and rat H9C2 cells to investigate the effect of mitochondrial injury on necroptosis in irradiated cardiomyocytes and to further elucidate the mechanism underlying radiation-induced heart disease and discover possible preventive targets. After γ-ray irradiation, the expression levels of necroptosis markers were increased, along with higher oxidative stress and mitochondrial injury. These effects could be abated by overexpression of protein tyrosine phosphatase, mitochondrial 1 (PTPMT1). Inhibiting oxidative stress or increasing the expression of PTPMT1 could protect against radiation-induced mitochondrial injury and then decrease the necroptosis of cardiomyocytes. These results suggest that PTPMT1 may be a new target for the treatment of radiation-induced heart disease.

**NEW & NOTEWORTHY** Effective strategies are still lacking for treating RIHD, with unclear pathological mechanisms. In cardiomyocytes model of radiation-induced injuries, we found γ-ray irradiation decreased the expression of PTPMT1, increased oxidative stress, and induced mitochondrial dysfunction and necroptosis in iPSC-CMs. ROS inhibition attenuated radiation-induced mitochondrial damage and necroptosis. PTPMT1 protected cardiomyocytes from necroptosis induced by γ-ray irradiation by alleviating mitochondrial injury. Therefore, PTPMT1 might be a potential strategy for treating RIHD.

## Graphical abstract

**Figure:**
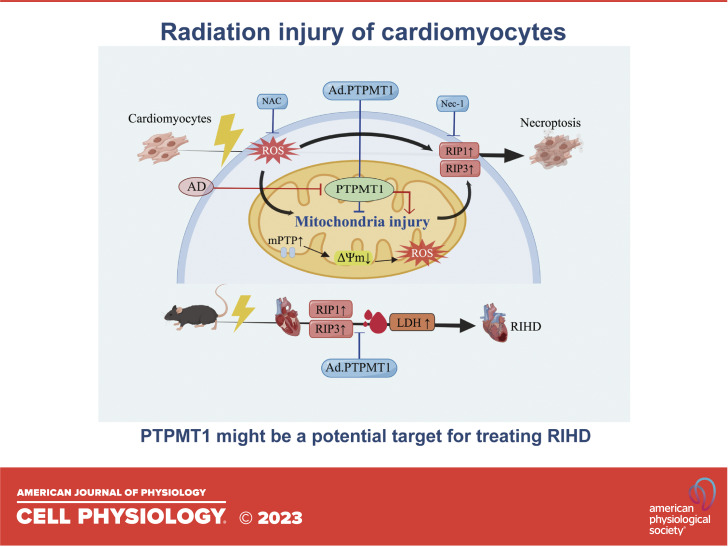

## INTRODUCTION

Radiation-induced heart disease (RIHD) is a serious side effect of thoracic radiotherapy and remains an effective treatment for many types of neoplasms (1). The deleterious effects of radiation on the heart are irreversible, causing coronary artery disease, valvular disease, myocardial disease with systolic and especially diastolic dysfunction, and conduction system abnormalities (2). Regarding the pathogenesis of RIHD, endothelial cells are typically considered the main damage-targeted cells (3). Some studies illustrate that myocardial degeneration and diffuse fibrosis are preceded by irradiation-induced damage to the capillary network (4). However, cardiomyocytes, the primary part both in the structure and function of the heart, have not been studied extensively in RIHD research because they are consistently deemed radiation insensitive. Therefore, the underlying mechanisms for radiation-induced injuries of cardiomyocytes are not clear.

Necroptosis is a type of regulated necrotic cell death driven by defined molecules (5). It has been reported that necroptosis is involved in severe cardiac pathological conditions, including myocardial infarction and ischemia/reperfusion injury, and plays an important role in myocardial remodeling and heart failure (6). Liu et al. (7) also reported that suppressing necroptosis with the receptor-interacting protein 1 (RIP1) inhibitor necrostatin-1 (Nec-1) protects against ischemia/reperfusion injury in a rat model. Cardiomyocyte mitochondria are critical to cardiac function, and many studies have reported that mitochondrial dysfunction and reactive oxygen species (ROS) production contribute to necroptosis development (8).

Protein tyrosine phosphatase, mitochondrial 1 (PTPMT1), a newly identified Pten-like mitochondrial phosphatase, is exclusively localized to the inner membrane of mitochondria in most tissues (9). PTPMT1 participates in the biosynthesis of cardiolipin, which is the essential element of the mitochondrial inner membrane, and downregulating PTPMT1 can inhibit the biosynthesis of cardiolipin, causing mitochondrial dysfunction (10, 11).

It is reasonable to ask whether necroptosis and PTPMT1 are involved in radiation-induced cardiomyocyte injury and whether they contribute to the progression of RIHD. In the present study, we investigated the effects of γ-ray irradiation on mitochondrial injury and necroptosis in two types of cardiomyocytes [induced pluripotent stem cell-derived cardiomyocytes (iPSC-CMs) and H9C2 cells] and the possible involvement of PTPMT1.

## METHODS AND MATERIALS

### iPSC-CMs and H9C2 Cells

The B1 hiPSC line was purchased from Cellapy (CA4025106, Beijing, China). The methods of cell culture, differentiation, and identification are detailed in the Supplemental Materials (all Supplemental Material is available at https://doi.org/10.6084/m9.figshare.21383700).

H9C2 cells were purchased from American Type Culture Collection (ATCC) and cultured in DMEM (C11965500, Gibco, Grand Island, NY) containing 10% FBS (F7524, Sigma, St. Louis, MO) and 1% HEPES (H1095, Solarbio, Beijing, China).

### Recombinant Viruses

Adenovirus-protein tyrosine phosphatase, mitochondrial 1 (Ad.PTPMT1) (VH877819), a recombinant adenovirus construct encoding the protein PTPMT1, and ADM-FH (VH88001), a recombinant adenovirus not carrying exogenous genes, were purchased from WZ Biosciences Inc. (Shandong, China). H9C2 cells were incubated in a medium with Ad.PTPMT1 at 150 multiplicity of infection (MOI) for 24 h to overexpress PTPMT1 before irradiation. AdV5-CMV-C-FH (ADM-FH) was used as a control adenovirus.

### Irradiation Procedure

Cells were irradiated with a single dose γ-ray of 10 and 20 Gy (68.31 cGy/min), and then the medium was replaced or added with Nec-1 (20 μM) (S8037, Selleck, Houston, TX), N-acetyl-l-cysteine (NAC) (20 μM) (S1623, Selleck), or Alexidine (AD, 50 μM) (1715-30-6, Yuanye Biotechnology, Shanghai, China) after irradiation. All these reagents were first dissolved in DMSO to 10 mM and then diluted in DMEM. Table 1 contains detailed information including validation references of these reagents.

**Table 1.** Reagents used in this study

Name	Company	Cat. No.	PMID
Nec-1	Selleck	S8037	35278352 34385713
NAC	Selleck	S1623	35738281 30290143

NAC, N-acetyl-l-cysteine; Nec-1, necrostatin-1.

### Western Blotting

The cells were collected for protein extraction and lysed with RIPA lysis buffer. buffer supplemented with premixed protease and phosphatase inhibitors. The protein concentration was determined by a bicinchoninic acid (BCA) protein assay kit (23227, Thermo Fisher Scientific, Waltham, MA). Then, 10% separating gel and 5% concentrated gel were prepared for SDS-PAGE. The protein samples of each group were fractionated by SDS-PAGE and transferred to PVDF membranes (Fisher Scientific, Hampton, NH) using Fast Wet Transfer System (eBlot L1, GenScript Biotech Corporation, Nanjing, China). Later, the membranes were blocked with blocking-specific buffer (C200501, Sunshine Yingrui Biotechnology, Beijing, China) for 15 min at room temperature, washed twice with 1× Tris-buffered saline Tween (TBST), snipped according to molecular weight and then incubated with primary antibodies against cysteinyl aspartate-specific proteinase 3 (Caspase3) (No. 9662), poly ADP-ribose polymerase (PARP) (No. 9542), receptor-interacting protein 1 (RIP1) (No. 3493), receptor-interacting protein 3 (RIP3) (No. 10188/No. 15828), phosphorylated-mixed lineage kinase domain-like (p-MLKL) (Ser358, No. 91689), phosphorylated-adenosine monophosphate-activated protein kinase (p-AMPK) (Thr172, No. 8208), phosphorylated-dynamin-related protein 1 (p-DRP1) (Ser616, No. 3455) (1:1,000, CST, Danvers, MA), phosphorylated-mitochondrial fission factor (p-MFF) (AF2365, 1:500, Affinity Biosciences), PTPMT1 (11493-1-AP, 1:500), and β-actin (81115-1-RR, 1:5,000) (Proteintech, Chicago, IL) overnight at 4°C. All the primary antibodies were used in the dilutions (P0023A, Beyotime Biotechnology, Shanghai, China). Thereafter, the membranes were washed three times and exposed to horseradish peroxidase (HRP)-conjugated goat anti-rabbit IgG (GB23303, Servicebio, Wuhan, China) (1:5,000 dilution with TBST) at room temperature for 1 h. Enhanced chemiluminescence (ECL) substrate working solution (0120528, Zoman Biotechnology Beijing, China) was prepared by mixing substrate A and B in equal volume, and then was covered to the snipped membranes. The fluorescent signal was detected using an imaging system (Tanon, Shanghai, China). Image J software (National Institutes of Health, Bethesda, MD) was used to quantify the integrated absorbance (IA) value of protein bands. Protein expression levels were normalized to β-actin protein expression, then the relative expression levels of the protein were normalized to the control group. Table 2 contains detailed information including validation references of primary antibodies and secondary antibodies.

**Table 2.** Antibodies used in this study

Name	Company	Cat. No.	Concentration	PMID
Caspase3	Cell Signaling	9662	1:1,000	36733226 36936401
PARP	Cell Signaling	9542	1:1,000	37046036 37024974
RIP1	Cell Signaling	3493	1:1,000	36715448 36774342
RIP3	Cell Signaling	10188	1:1,000	36470869 36604411
RIP3	Cell Signaling	15828	1:1,000	36875528 34360749
p-MLKL	Cell Signaling	91689	1:1,000	36060138 35657206
p-MLKL	Cell Signaling	37333	1:1,000	36333786 35587515
p-AMPK	Cell Signaling	8208	1:1,000	35182712 34439305
p-DRP1	Cell Signaling	3455	1:1,000	36327794 36168629
p-MFF	Affinity Biosciences	AF2365	1:500	36478988 35383983
PTPMT1	Proteintech	11493-1-AP	1:500	36575176 33503428 21986498
β-Actin	Proteintech	81115-1-RR	1:5,000	36731870 32781474 36077440
HRP-conjugated goat anti-rabbit IgG	Servicebio	GB23303	1:5,000	34815544 34819088

Caspase3, cysteinyl aspartate-specific proteinase 3; HRP, horseradish peroxidase; PARP, poly ADP-ribose polymerase; p-AMPK, phosphorylated-adenosine monophosphate-activated protein kinase; p-DRP1, phosphorylated-dynamin-related protein 1; p-MFF, phosphorylated-mitochondrial fission factor; p-MLKL, phosphorylated-mixed lineage kinase domain-like; PTPMT1, protein tyrosine phosphatase, mitochondrial 1; RIP1, receptor-interacting protein 1; RIP2, receptor-interacting protein 2; RIP3, receptor-interacting protein 3.

### Real-Time PCR Analysis of Gene Expression

Total RNA was isolated from cardiomyocytes using the RNA-Quick Purification Kit (RN001, Yishan Biotechnology, Shanghai, China), all RNA samples were treated with DNase I, and complementary DNA (cDNA) was synthesized by using the cDNA Reverse Transcription Kit (RT001, Yishan Biotechnology) according to the manufacturer’s protocol. The mRNA expression of RIP1, RIP3, calmodulin-dependent protein kinase II (CAMK II), PTPMT1, and AMPK was quantified using 2 × RealStar Power SYBR qPCR Mix (High ROX) (A311-10, Genstar, Beijing, China) on a 7500 real-time PCR system (ABI, Foster City, CA). The relative expression levels were calculated by the 2^−ΔΔCT^ method using β-actin as the control. The primers are listed in Table 3.

**Table 3.** The primer sequence for qPCR

Gene	Sequence
RIP1-F	CTGGTGTCTTGGGCTGATA
RIP1-R	GGGCAGGGATGCTACTAAA
RIP3-F	CCGGAGCCAAATCCAGTAACA
RIP3-R	GCTTCAGGATCTTTAGGGCCTTC
CAMK II-F	GCTGGGAGAAGAGGCAA
CAMK II-R	ACGAAACCCTGTGGTGAA
PTPMT1-F	GGTGGCAGCATACCTGAT
PTPMT1-R	CTTGGCGATGGCTCTTAC
AMPK-F	GCTTTTCAGGCATCCTCAT
AMPK-R	CATCCAGCCTTCCATTCTT
β-Actin-F	GGGACCTGACTGACTACCTC
β-Actin-R	CTTAATGTCACGCACGATT

### Analysis of Intracellular ROS Production

The 5-(and 6-)chloromethy-2,7-dichlorodihydrofluorescein diacetate, acetyl ester (CM-H2DCFDA) probe (C6827, Invitrogen, Carlsbad, CA) was used to investigate the generation of ROS in cardiomyocytes. Pretreated cells were incubated in serum-free medium containing 10 μM CM-H2DCFDA for 30 min at 37°C in the dark. Thereafter, the cells were trypsinized for flow cytometry analysis (BD Biosciences, Franklin Lakes, NJ) or observed in situ using a fluorescence confocal microscope (CrestOptics, Italy). Green fluorescence intensity was proportional to ROS content. The mean fluorescence intensity (MFI) was quantified by ImageJ software (National Institutes of Health, Bethesda, MD).

### Determination of Mitochondrial ROS

The fluorescent probe mitochondrial superoxide (MitoSOX) (M36008, Invitrogen) was used according to the manufacturer’s protocol. Briefly, cells were incubated with 5 μM MitoSOX in culture medium for 10 min, followed by three washes with prewarmed PBS. Cells were observed using the fluorescence confocal microscope. For flow cytometry analysis, after loading of MitoSOX, cells were collected by trypsinization and washed in PBS, and measurements were performed using FACS calibur system (BD Biosciences). MitoSOX red was excited by laser at 488 nm, and the data were collected at 585/42 nm channel. The data were presented by histogram of mean intensity of MitoSOX fluorescence.

### Analysis of Mitochondrial Membrane Potential

The mitochondrial membrane potential (ΔΨm) of cardiomyocytes was measured by the cationic probe JC-1 (C2006, Beyotime Biotechnology, Shanghai, China), which aggregates in the mitochondrial matrix of normal cells, showing red fluorescence, and turns into monomers, showing green fluorescence as ΔΨm decreases. The cells were trypsinized, stained with JC-1 at 37°C in the dark for 20 min, and then detected by flow cytometry. In addition, the red and green fluorescence was determined in situ using a fluorescence confocal microscope. The mean fluorescence intensity (MFI) was quantified by ImageJ software, and ΔΨm was calculated as the MFI ratio of red versus green.

### Analysis of Mitochondrial Permeability Transition Pore Opening

The opening of mitochondrial permeability transition pore (mPTP) in cardiomyocytes was detected using a mPTP assay kit (GMS10095.1, Genmed Scientifics Inc, Arlington, MA) according to the manufacturer’s protocol. The cells were rinsed with prewarmed wash buffer, incubated with staining buffer at 37°C in the dark for 20 min, and then rinsed twice. The green fluorescence was detected using a confocal microscope. The MFI was quantified by ImageJ software and was negatively correlated with mPTP opening.

### Statistical Analysis

All data were analyzed with one-way analysis of variance (ANOVA) followed by Bonferroni post hoc tests using GraphPad Prism software version 5 and expressed as the means ± SE. Differences between treated and control groups were considered significant at a two-sided *P* < 0.05.

## RESULTS

### The Features and Morphology of iPSC-CMs

To better simulate the damage of irradiation on the heart in vitro, we not only performed experiments in the H9C2 myocardial cell line but also carried out assays with induced pluripotent stem cell-derived cardiomyocytes (iPSC-CMs). The morphology of cultured iPSC-CMs was observed by inverted microscopy. iPSC-derived CMs started spontaneous contraction after 10 days of differentiation. The digested, replated, and cultured iPSC-CMs grew as a monolayer, showing a clear cell shape on a Matrigel-coated plate (Supplemental Fig. S1*A*), and were positively stained by the CM-specific markers troponin T and sarcomeric actinin (Supplemental Fig. S1*B*) at *day 20* after differentiation.

### γ-Ray Irradiation Can Induce Necroptosis in iPSC-CMs

**Figure 1.**
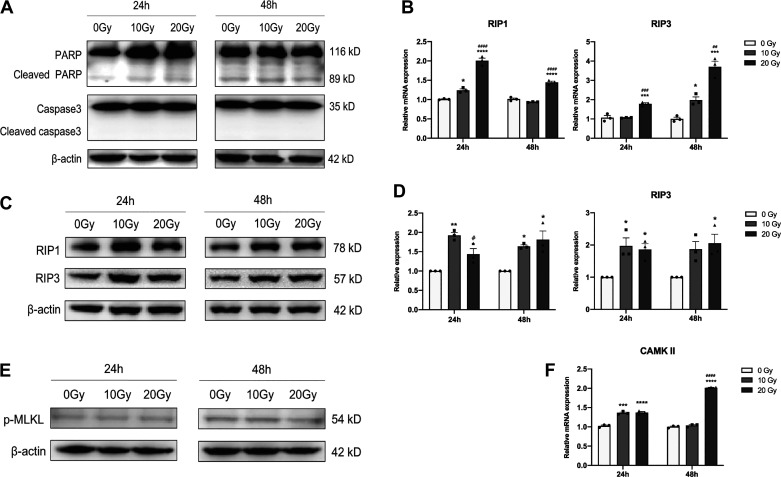
Effects of γ-ray irradiation on apoptosis and necroptosis in iPSC-CMs. *A*: Western blotting was used to determine the expression of PARP, cleaved PARP, caspase-3, and cleaved caspase-3. qPCR (*B*) and Western blotting (*C*) were used to determine the expression of RIP1 and RIP3. *D*: the histogram shows the quantitative results of *C*. *E*: Western blotting was used to determine the expression of p-MLKL. *F*: qPCR was used to determine the gene expression of CAMK II. Data are expressed as the means ± SE, *n* = 3 replicates. One-way ANOVA was used for statistical analysis. *P < 0.05, **P < 0.01, ***P < 0.001, ****P < 0.0001, vs. 0 Gy group; #*P* < 0.05, ##*P* < 0.01, ###*P* < 0.001, ####*P* < 0.0001, vs. 10 Gy group. CAMK II, calmodulin-dependent protein kinase II; iPSC-CMs, induced pluripotent stem cell-derived cardiomyocytes; PARP, poly ADP-ribose polymerase; p-MLKL, phosphorylated-mixed lineage kinase domain-like; RIP1, receptor-interacting protein 1; RIP2, receptor-interacting protein 2.

Related protein markers were detected to investigate whether apoptosis or necroptosis occurred in iPSC-CMs after γ-ray irradiation. The Western blotting results showed that there were no obvious changes in PARP and Caspase3 expression in irradiated iPSC-CMs (Fig. 1*A*). However, necroptosis-related RIP1 and RIP3 were significantly increased in irradiated iPSC-CMs compared with control cells at both the gene (Fig. 1*B*) and protein levels (Fig. 1, *C* and *D*). We did not observe a significant increase in p-MLKL expression (Fig. 1*E*), but the qPCR results (Fig. 1*F*) showed that compared with unirradiated cells, the expression of CAMK II was significantly increased in irradiated iPSC-CMs. On the other hand, the effect of γ-ray irradiation on cell viability in cardiomyocyte was conducted. The results of cell counting kit-8 (CCK-8) assay showed that, compared with the unirradiated group, the survival rates were reduced in 20 Gy group at 24 h, 48 h, and 72 h after irradiation, and in 5 Gy group or 10 Gy group at 48 h and 72 h after irradiation (Supplemental Fig. S2).

### γ-Ray Irradiation Induced Oxidative Stress and Mitochondrial Injury in iPSC-CMs

**Figure 2.**
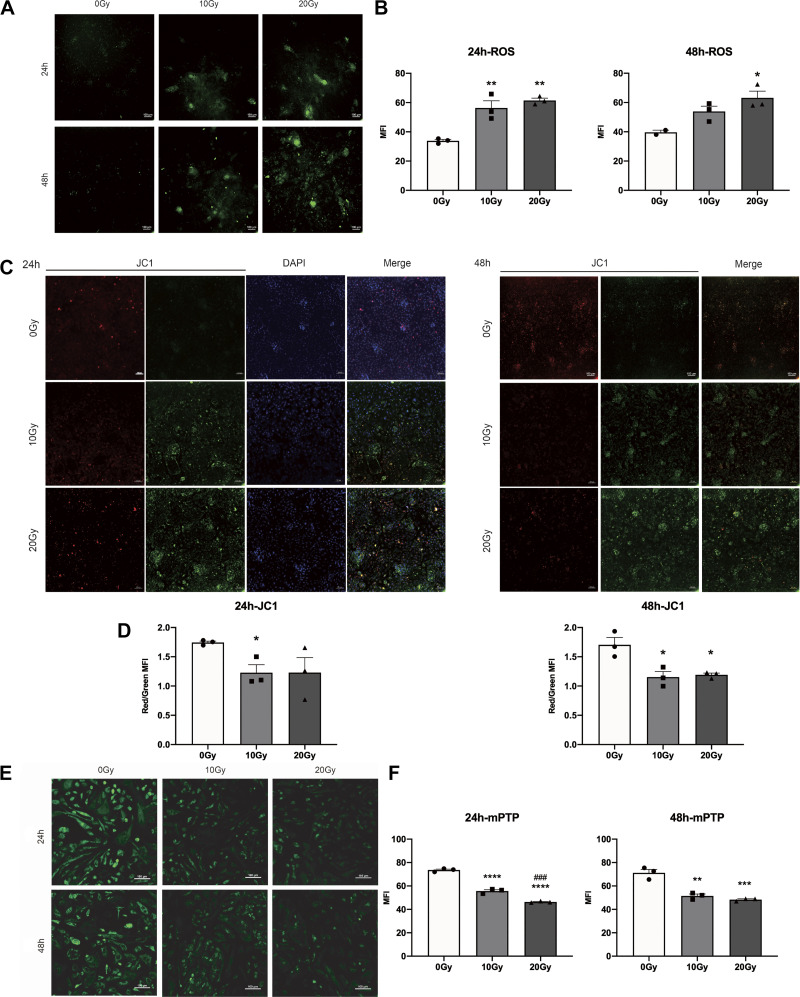
Effects of γ-ray irradiation on oxidative stress and mitochondrial injury in iPSC-CMs. A confocal microscope was used to detect ROS production (*A*), and the histogram shows the quantitative results (*B*). Representative confocal microscopy images showing the ΔΨm (*C*), and the histogram shows the quantitative results (*D*). Representative confocal microscopy images showing mPTP opening (*E*), and the histogram shows the quantitative results (*F*). Data are expressed as the means ± SE, *n* = 3 replicates. One-way ANOVA was used for statistical analysis. *P < 0.05, **P < 0.01, ***P < 0.001, ****P < 0.0001, vs. control group (0 Gy group); ###*P* < 0.001, vs. 10 Gy group. Scale bars = 100 μm. iPSC-CMs, induced pluripotent stem cell-derived cardiomyocytes; mPTP, mitochondrial permeability transition pore; ΔΨm, mitochondrial membrane potential.

Activation of oxidative stress and mitochondrial injury plays vital roles in many irradiation-induced diseases, as well as in the development of cardiac dysfunction under various pathological conditions. We irradiated iPSC-CMs as described earlier and assessed intracellular ROS production, ΔΨm, and the opening of mPTP in iPSC-CMs at the indicated time points. The results showed that the MFI of ROS increased with increasing irradiation time and dose (Fig. 2, *A* and *B*). The JC-1 results showed that the ratio of fluorescence intensity (red/green MFI) was significantly decreased in irradiated iPSC-CMs compared with unirradiated cells, indicating mitochondrial depolarization and a decrease in ΔΨm in iPSC-CMs (Fig. 2, *C* and *D*). Confocal results also showed that the MFI of mPTP was significantly decreased in irradiated iPSC-CMs, suggesting an increase in the opening of mPTP (Fig. 2, *E* and *F*). Furthermore, we detected the mitochondrial ROS in H9C2 cells after irradiation. The flow cytometry results showed that the production of mitochondrial ROS was significantly increased in irradiated groups compared with the control group (Supplemental Fig. S3, *A* and *B*). Similar results were observed in the fluorescence confocal experiment (Supplemental Fig. S3, *C* and *D*).

### Effects of γ-Ray Irradiation on AMPK, DRP1, and MFF Signaling in iPSC-CMs

**Figure 3.**
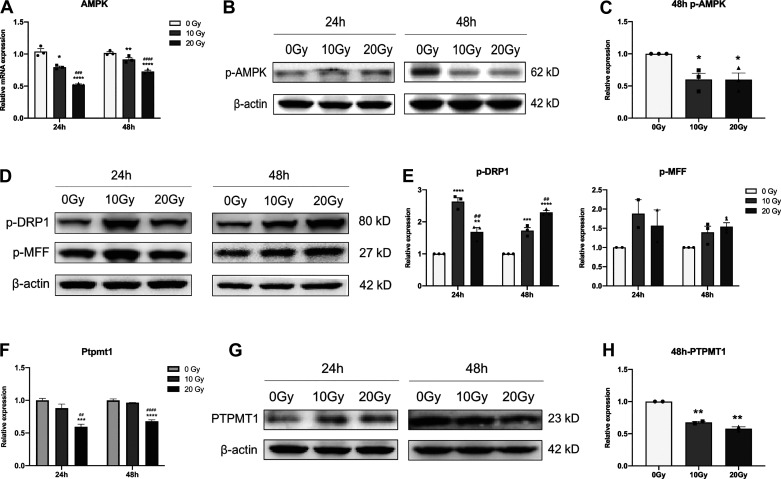
Effects of γ-ray irradiation on AMPK, DRP1 and MFF signaling and changes in PTPMT1 expression in iPSC-CMs. qPCR (*A*) and Western blotting (*B*) were used to determine the expression of AMPK, and the histogram shows the quantitative results of *B* (*C*). Western blotting was used to determine the expression of fission-related proteins (p-DRP-1 and p-MFF; *D*), and the quantitative results are shown (*E*). qPCR (*F*) and Western blotting (*G*) were used to determine the gene expression level of PTPMT1, and the quantitative results of *G* are shown (*H*). Data are expressed as the means ± SE, *n* = 3 (replicates). One-way ANOVA was used for statistical analysis. *P < 0.05, **P < 0.01, ***P < 0.001, ****P < 0.0001, vs. 0 Gy group; ##*P* < 0.01, ###*P* < 0.001, ####*P* < 0.0001, vs. 10 Gy group. iPSC-CMs, induced pluripotent stem cell-derived cardiomyocytes; p-DRP1, phosphorylated-dynamin-related protein 1; p-MFF, phosphorylated-mitochondrial fission factor; PTPMT1, protein tyrosine phosphatase, mitochondrial 1.

To detect whether γ-ray irradiation affects the energy homeostasis or fission of mitochondria, we measured the expression levels of related molecules. qPCR analysis showed decreased expression of AMPK in irradiated iPSC-CMs (Fig. 3*A*), and Western blotting showed that the expression of p-AMPK significantly decreased in irradiated iPSC-CMs 48 h after irradiation compared with unirradiated cells (Fig. 3, *B* and *C*). Furthermore, the results showed that compared with that in unirradiated cells, p-DRP1 and p-MFF expression were significantly increased in irradiated iPSC-CMs (Fig. 3, *D* and *E*).

### γ-Ray Irradiation Decreased the Expression of PTPMT1 in iPSC-CMs

To further explore the underlying mechanisms of γ-ray irradiation-induced mitochondria injury in iPSC-CMs, the expression of PTPMT1 was measured. qPCR analysis showed that the expression of PTPMT1 significantly decreased in irradiated iPSC-CMs after irradiation with 20 Gy (Fig. 3*F*), and Western blotting analyses showed that the expression of PTPMT1 significantly decreased in irradiated iPSC-CMs 48 h after irradiation compared with unirradiated cells (Fig. 3, *G* and *H*), implying that PTPMT1 may participate in the effect of γ-ray irradiation-induced mitochondrial injury in iPSC-CMs.

### Nec-1 and NAC Can Attenuate γ-Ray Irradiation-Induced Cardiomyocyte Oxidative Stress and Mitochondrial Injury

**Figure 4.**
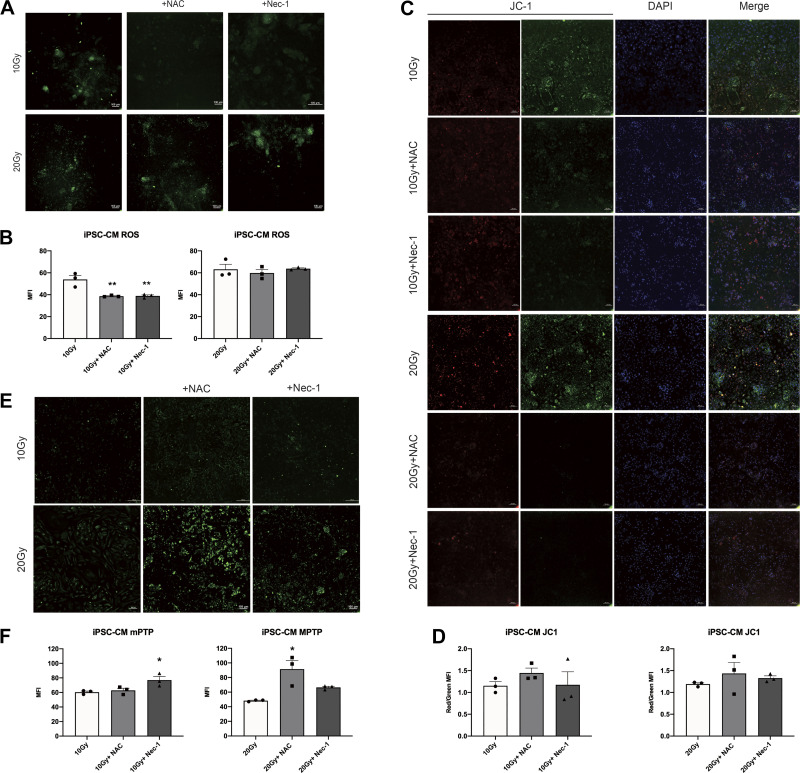
Effects of NAC or Nec-1 on oxidative stress and mitochondrial injury in iPSC-CMs irradiated by γ-ray. At 48 h after irradiation, confocal microscopy was used to detect ROS production in iPSC-CMs treated with NAC or Nec-1 (*A*), and the histogram shows the quantitative results (*B*). Representative confocal microscopy images showing the ΔΨm (*C*), and the quantitative results (*D*). Representative confocal microscopy images showing mPTP opening (*E*), and the quantitative results (*F*). Data are expressed as the means ± SE, *n* = 3 replicates. One-way ANOVA was used for statistical analysis. *P < 0.05, **P < 0.01, vs. control group (drugs untreated group); Scale bars = 100 μm. iPSC-CMs, induced pluripotent stem cell-derived cardiomyocytes; mPTP, mitochondrial permeability transition pore; NAC, N-acetyl-l-cysteine; Nec-1, necrostatin-1; ROS, reactive oxygen species; ΔΨm, mitochondrial membrane potential.

To explore the relationship among oxidative stress, mitochondrial injury, and necroptosis in cardiomyocytes, we treated iPSC-CMs and H9C2 cells with Nec-1 or NAC after irradiation. The results of intracellular ROS production showed that the MFI was significantly decreased in 10 Gy-irradiated iPSC-CMs after treatment with NAC or Nec-1 compared with cells irradiated alone (Fig. 4, *A* and *B*). For ΔΨm, an increase in red fluorescence intensity and a decrease in green fluorescence intensity were observed in 10 Gy-irradiated iPSC-CMs after treatment with NAC or Nec-1 (Fig. 4, *C* and *D*). The opening of mPTP displayed an enhanced trend in MFI in irradiated iPSC-CMs after treatment, and the difference reached statistical significance in 10 Gy-irradiated iPSC-CMs after treatment with Nec-1 and in 20 Gy-irradiated iPSC-CMs after treatment with NAC, compared with cells irradiated alone (Fig. 4, *E* and *F*).

In 20 Gy-irradiated H9C2 cells, the flow cytometry results showed that Nec-1 and NAC reduced the peak value of intracellular ROS, shifted the curve to the left, and decreased the MFI (Supplemental Fig. S4, *A* and *B*), meanwhile, NAC treatment significantly reduced the production of mitochondrial ROS in 20 Gy-irradiated H9C2 cells (Supplemental Fig. S4, *C* and *D*). Compared with cells irradiated alone, the proportion of red/green subsets increased significantly after adding NAC and Nec-1 to irradiated H9C2 cells (Supplemental Fig. S4, *E* and *F*). Similar results were observed in the fluorescence confocal experiment (Supplemental Fig. S4*G*). In our assays for the opening of mPTP, an enhanced trend in MFI was observed in irradiated H9C2 cells after treatment with NAC or Nec-1 compared with cells irradiated alone (Supplemental Fig. S4*H*). Moreover, the Western blotting results showed that NAC reduced the expression levels of RIP1, RIP3, and p-MLKL more strongly than Nec-1 (Supplemental Fig. S4*I*).

### Inhibition of PTPMT1 Expression Can Induce Oxidative Stress and Mitochondrial Dysfunction in Cardiomyocytes

**Figure 5.**
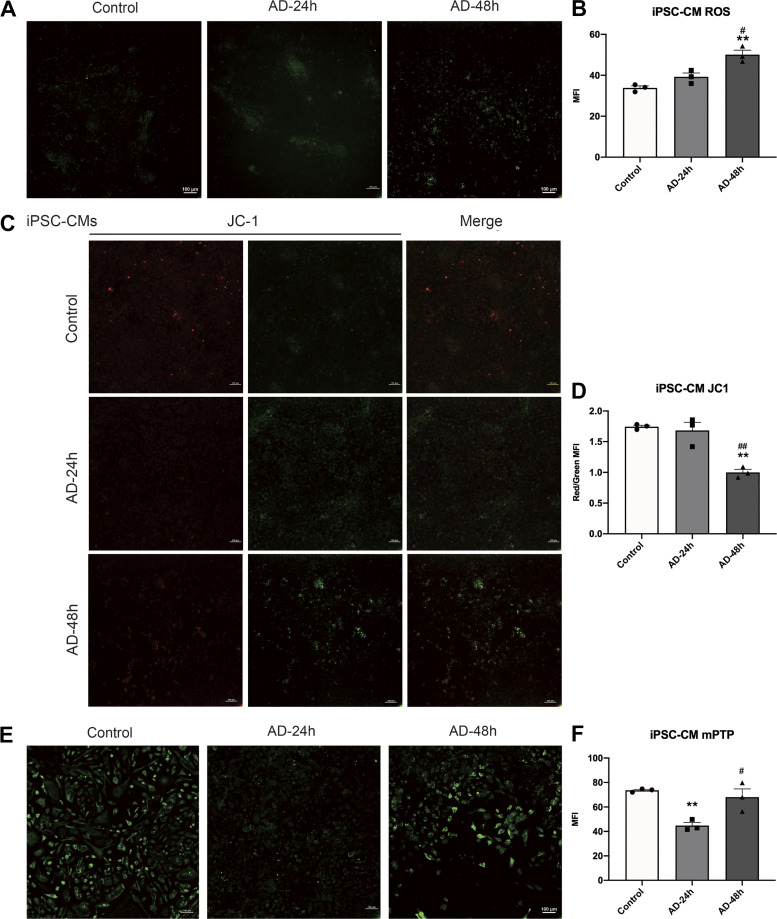
Effects of AD on oxidative stress and mitochondrial injury in iPSC-CMs. At 24 and 48 h after AD treatment, confocal microscopy was used to detect ROS production in iPSC-CMs (*A*), and the histogram shows the quantitative results (*B*). A confocal microscope was used to detect ΔΨm (*C*), and the quantitative results are shown (*D*). A confocal microscope was used to detect mPTP opening (*E*), and the histogram shows the quantitative results (*F*). Data are expressed as the means ± SE, *n* = 3 replicates. One-way ANOVA was used for statistical analysis. **P < 0.01, vs. control group (drugs untreated group); #*P* < 0.05, ##*P* < 0.01, vs. AD-24 h group. Scale bars = 100 μm. AD, Alexidine; iPSC-CMs, induced pluripotent stem cell-derived cardiomyocytes; mPTP, mitochondrial permeability transition pore; ROS, reactive oxygen species; ΔΨm, mitochondrial membrane potential.

Given the possibility that PTPMT1 plays a vital role in the effect of γ-ray irradiation-induced cardiomyocyte injury, we treated iPSC-CMs with AD. We found that intracellular ROS production was increased at 24 h and reached statistical significance at 48 h after treatment with AD compared with the control group (Fig. 5, *A* and *B*). JC-1 fluorescence of iPSC-CMs showed that ΔΨm was significantly decreased at 24 h and 48 h after treatment with AD compared with the control group (Fig. 5, *C* and *D*). The opening of mPTP was significantly decreased at 24 h after treatment and returned to normal levels at 48 h (Fig. 5, *E* and *F*).

Furthermore, we performed similar experiments in H9C2 cells and the results showed that AD treatment inhibited PTPMT1 expression in a dose-dependent manner (Supplemental Fig. S5*A*). It showed that intracellular ROS was significantly increased after treatment for 48 h (Supplemental Fig. S5, *B* and *C*), and moreover, the increase of mitochondrial ROS production was observed after 24 h after AD treatment (Supplemental Fig. S5, *D*–*G*). AD also induced a reduction in ΔΨm 48 h after radiation (Supplemental Fig. S5, *H* and *I*) and increased the opening of mPTP at 24 h and 48 h after radiation (Supplemental Fig. S5, *J* and *K*).

### Overexpression of PTPMT1 Attenuated Irradiation-Induced Mitochondrial Injury in Cardiomyocytes

**Figure 6.**
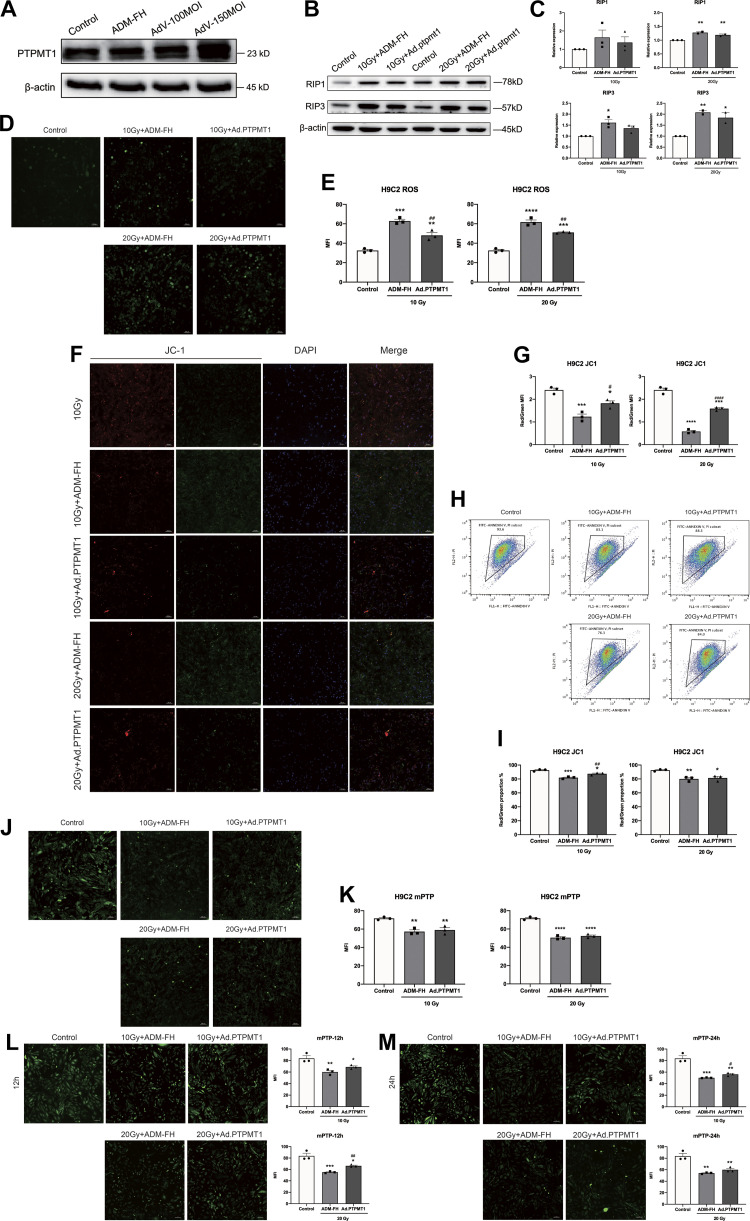
Effects of Ad.PTPMT1 on oxidative stress and mitochondrial injury in H9C2 cells. Western blotting was carried out to determine the expression of PTPMT1 protein in H9C2 cells treated with ADM-FH (negative control), 100 MOI Ad.PTPMT1, and 150 MOI Ad.PTPMT1. At 48 h after γ-ray irradiation (*A*), Western blotting was used to determine the expression of RIP3 (*B*), and the quantitative results are shown (*C*). Confocal microscopy was used to detect ROS production (*D*), and the histogram shows the quantitative results (*E*). Representative confocal microscopy images showing the ΔΨm (*F*) and the quantitative results (*G*). Flow cytometry was used to measure ΔΨm (*H*), and the statistical results are shown (*I*). Representative confocal microscopy images showing mPTP opening (*J*) and the quantitative results (*K*). Representative confocal microscopy images showing mPTP opening and the quantitative results at 12 h (*L*) and 24 h (*M*) after γ-ray irradiation. Data are expressed as the means ± SE, *n* = 3 replicates. One-way ANOVA was used for statistical analysis. *P < 0.05, **P < 0.01, ***P < 0.001, ****P < 0.001, vs. the control group (unirradiated H9C2 group); #*P* < 0.05, ##*P* < 0.01, ####*P* < 0.0001, vs. ADM-FH group. Scale bars = 100 μm. ADM-FH, AdV5-CMV-C-FH; MOI, multiplicity of infection; mPTP, mitochondrial permeability transition pore; PTPMT1, protein tyrosine phosphatase, mitochondrial 1; RIP3, receptor-interacting protein 3; ΔΨm, mitochondrial membrane potential.

To further investigate the effect of overexpression of PTPMT1 on irradiation-induced injury in cardiomyocytes, we generated PTPMT1-overexpressing cardiomyocytes using Ad.PTPMT1 to infect H9C2 cells. We treated H9C2 cells with Ad.PTPMT1, and ADM-FH was used as a virus-negative control. The Western blotting results showed that PTPMT1 expression in Ad.PTPMT1-treated H9C2 cells was significantly increased compared with that in the control group (Fig. 6*A*). Then, pretreated H9C2 cells were irradiated, and the relative assays were performed 48 h after irradiation.

In the assays, Western blotting results showed that Ad.PTPMT1 reduced the expression levels of RIP3 (Fig. 6, *B* and *C*). The confocal fluorescence analysis showed that (Fig. 6, *D* and *E*) intracellular ROS production was clearly increased after irradiation and decreased significantly after Ad.PTPMT1 treatment. ΔΨm was obviously decreased after irradiation and increased significantly after Ad.PTPMT1 treatment (Fig. 6, *F* and *G*). The flow cytometry results (Fig. 6, *H* and *I*) were consistent with the results of the confocal fluorescence analysis. At 48 h after irradiation, the protective effect of mPTP opening was not observed in H9C2 cells overexpressing PTPMT1 (Fig. 6, *J* and *K*). However, subsequent experiments showed that Ad.PTPMT1 treatment significantly improved the MFI of mPTP in irradiated H9C2 cells at 12 h and 24 h after irradiation, compared with that in negative control groups (Fig. 6, *L* and *M*).

### Effects of Ad.PTPMT1 on AMPK, DRP1, and MFF Signaling in Cardiomyocytes

To detect the effect of overexpression of PTPMT1 on the energy homeostasis or fission of mitochondria, H9C2 cells were divided into three groups (unirradiated group, 20 Gy-irradiated plus ADM-FH treated group, and 20 Gy-irradiated plus Ad.PTPMT1 treated group), and Western blotting was carried out to determine the expression of P-AMPK, P-DRP1, and P-MFF. The results showed decreased expression of p-AMPK and increased expression of p-MFF and p-DRP1 in 20 Gy-irradiated plus ADM-FH treated group, interestingly, treatment with Ad.PTPMT1 could reverse these changes induced by irradiation (Supplemental Fig. S6, *A* and *B*).

Furthermore, transmission electron microscopy was used to observe whether PTPMT1 overexpression can play a protective effect on mitochondrial structure in irradiated H9C2 cells. The results of electron microscopy observation showed that irradiation could damage the mitochondrial structure in 20 Gy-irradiated plus ADM-FH-treated group manifested as mitochondrial swelling, destruction of internal structures and the specific structural ridges was unclear. By contrast, PTPMT1 overexpression could alleviate these damages, manifested as the mitochondrial structure was more intact and the specific structural ridges were more clear (Supplemental Fig. S7).

## DISCUSSION

The advent of human iPSC technology provides an unprecedented opportunity to generate human patient-specific cells that can be used for disease modeling, personalized drug screening, and regenerative medicine for precision medicine (12, 13). Currently, the ability to differentiate iPSCs into disease-relevant cell types, such as iPSC-CMs, is increasingly being refined (14). The implementation of this unique and clinically relevant model system has significant advantages in cardiovascular research because it circumvents the complexity of transforming data in models of different species and biological characteristics. In contrast to many previous methods, the cardiomyocyte differentiation protocol described here does not require cell aggregation or the addition of activin A or bone morphogenetic protein 4 (BMP4) (15) and robustly generates cell cultures that are highly positive for cardiac troponin T and sarcomeric α-actinin.

Recent advances have shown that necroptosis is involved in the development of cardiovascular diseases (16). In a rat model of ischemia/reperfusion, necroptosis played a vital role in disease development, and inhibition of necroptosis caused an increase in left ventricular systolic pressure, which produced a positive inotropic effect (17). In the experiment, elevated expression levels of RIP1 and RIP3 were detected in irradiated iPSC-CMs, which indicated the occurrence of necroptosis of cardiomyocytes. What perplexed us was that we did not observe a significant increase in p-MLKL expression, which is typically considered the direct substrate of RIP3 and the effector in the necroptosis process. However, new evidence demonstrates that CaMKII is also a key mediator of RIP3-induced myocardial necroptosis and apoptosis via the regulation of mPTP opening (18). The qPCR results showed that the expression of CAMK II was significantly increased in iPSC-CMs after irradiation, suggesting that CAMK II may be an important downstream molecule in the process of γ-ray-induced necroptosis.

The focus on radiation-induced mitochondrial dysfunction is increasing. An in vivo rat model of local heart irradiation revealed that irradiation could result in mitochondrial dysfunction, with an increase in the opening of the mPTP and a decrease in ΔΨm (19). Ionizing radiation damages mitochondria and impairs mitochondrial respiration, leading to the overproduction of mitochondrial ROS. Mitochondria are the sites of intracellular ROS generation through mitochondrial oxidative phosphorylation (OXPHOS) (20). mPTP opening is a mitochondrial response to an oxidative challenge resulting in an amplified ROS signal, which, depending on ROS levels, may result in different outcomes. At higher ROS levels, longer mPTP openings may release a ROS burst leading to destruction of mitochondria, and if propagated from mitochondrion to mitochondrion, of the cell itself (21). Our results indicated that γ-ray irradiation induced oxidative stress and mitochondrial injury in iPSC-CMs, including an increase in the opening of the mPTP, a decrease in ΔΨm, and the activation of ROS production. Moreover, our findings showed that the expression of AMPK significantly decreased and the expression of DRP1 and MFF significantly increased in irradiated iPSC-CMs, implying that γ-ray irradiation can impair the energy homeostasis of mitochondria and induce mitochondrial fragmentation and dysfunction in cardiomyocytes.

Studies have shown that the occurrence of necroptosis is closely related to mitochondrial dysfunction and elevated levels of oxidative stress (8). In our research, NAC and Nec-1 were used to treat irradiated iPSC-CMs and H9C2 cells. The results showed that NAC significantly inhibited the production of ROS and the opening of mPTP and inhibited the reduction of ΔΨm in irradiated cardiomyocytes. Moreover, the expression level of RIP1-RIP3-MLKL signaling molecules showed a downward trend after NAC treatment, suggesting that inhibition of ROS and ROS-induced mitochondrial damage can protect cells from necroptosis. Consistent with these results, increased ROS levels have been demonstrated to promote the stabilization of the RIP1/RIP3 necrosome (22). The latter study also provided evidence that RIP1 and RIP3 stimulate ROS production, which further promotes necrosome stabilization (23). In turn, inhibition of RIP1 (Nec-1 treatment) led to feedback inhibition of ROS production, which contributed to the maintenance of ΔΨm but had no significant effect on mPTP opening. Therefore, we postulated a radioreactive chain event of “ROS-mitochondrial damage-necroptosis” in cardiomyocytes.

PTPMT1 responds to nutrient and oxidative stress (24). Recent studies revealed that PTPMT1 is required for embryonic cardiac cardiolipin biosynthesis to regulate mitochondrial morphogenesis and heart development (25). Considering the close connection between necroptosis, mitochondrial function, and PTPMT1, we investigated whether PTPMT1 plays an important role in irradiation-induced cardiomyocyte damage. As expected, we detected a reduction in PTPMT1 at the gene and protein expression levels in irradiated iPSC-CMs. We also noticed that the PTPMT1 expression in protein level had no statistical significance with that of unirradiated group at 24 h, which is inconsistent with that of mRNA. We speculated that the response time of PTPMT1 protein was later than that of nucleic acid may be due to protein translation needing more time than that of mRNA transcript. But the exact reason needs to be further studied. Afterward, we investigated the role of PTPMT1 in mitochondrial damage and necroptosis by adding inhibitors or infecting cells with Ad.PTPMT1. Research has shown that PTPMT1 inhibition increases ROS levels and that oxidative damage dampens the release of FoxO1 from mitochondria (26). We found that inhibition of PTPMT1 caused a marked increase in mPTP opening in iPSC-CMs and H9C2 cells at 24 h after irradiation, induced ROS production, and decreased ΔΨm at 48 h after irradiation. Furthermore, overexpression of PTPMT1 significantly reduced ROS production and maintained ΔΨm in H9C2 cells but had no protective effect on mPTP opening at 48 h after irradiation. We hypothesized that the effect of Ad.PTPMT1 on mPTP may be rapid and transient, and this protective effect was not observed in the late stage of irradiation, but it had a sustained protective effect on the downstream effects of mPTP opening, including ROS production and changes in ΔΨm. Subsequently, we determined the changes in mPTP in PTPMT1-overexpressing cardiomyocytes at the early postirradiation stage to verify our findings. Thus, we speculated that the opening of mPTP appeared to be the first reactive step in the process of PTPMT1 disruption, followed by signal amplification of ROS and reduction of ΔΨm.

Based on the results obtained from in vitro experiments by using iPSC-CMs and H9C2 cells, we conducted in vivo animal experiments to validate these findings. Male C57BL/6 mice received single-dose irradiation with 20 Gy of γ rays locally to the chest, then irradiated mice were treated with necroptosis inhibitors Nec-1 (2 mg/kg) or Ad.PTPMT1 (5 × 10^8^ pfu), the cardiac tissue and peripheral serum samples were collected, and relative experiments were conducted. As shown in Supplemental Fig. S8, *A* and *B*, the results of Western blotting indicated that intraperitoneal injection of Nec-1 or intravenous injection of the Ad.PTPMT1 could downregulate the expression of p-MLKL, RIP1, and RIP3 induced by irradiation. The lactate dehydrogenase (LDH) detection results also suggested that treatment with Nec-1 and Ad.PTPMT1 could decrease the concentration of LDH in the serum induced by irradiation (Supplemental Fig. S8, *C* and *D*). These results preliminary validated the results in vitro and implied that administration of Nec-1 or overexpression of PTPMT1 could protect cardiomyocytes from irradiation-induced necroptosis.

### Conclusions

We demonstrated that γ-ray irradiation decreased the expression of PTPMT1, increased oxidative stress, and induced mitochondrial dysfunction and necroptosis in iPSC-CMs. PTPMT1 protected cardiomyocytes from necroptosis induced by γ-ray irradiation by alleviating mitochondrial injury. Our findings provide new insights into the mechanism of radiation damage to cardiomyocytes and will contribute to the discovery of new targets for the prevention and treatment of RIHD.

## DATA AVAILABILITY

Research data are stored in an institutional repository and will be shared upon request to the corresponding author.

## SUPPLEMENTAL DATA

10.6084/m9.figshare.21383700 Supplemental Figs. S1–S8: https://doi.org/10.6084/m9.figshare.21383700.

## GRANTS

This work was supported by the Scientific Research Project (NO. AWS21J003).

## DISCLOSURES

No conflicts of interest, financial or otherwise, are declared by the authors.

## AUTHOR CONTRIBUTIONS

Y.T. and H.W. conceived and designed research; J.Y., L.Y., Y.Z., N.T., H.D., and L.L. performed experiments; J.Y., Y.Z., N.T., H.D., L.L., and H.W. analyzed data; J.Y., L.Y., Y.Z., Y.T., and H.W. interpreted results of experiments; J.Y., L.Y., Y.Z., and H.W. prepared figures; J.Y. and Y.Z. drafted manuscript; Y.T. and H.W. edited and revised manuscript; H.W. approved final version of manuscript.
